# Genetic Characterization of the Arabian Horse Population in Tunisia Using Microsatellites

**DOI:** 10.3390/life15121925

**Published:** 2025-12-16

**Authors:** Mariem Jlassi, Iheb Dhifalli, Hatem Ouled Ahmed, Faten Lasfar, Mohamed El Gtari, Bayrem Jemmali

**Affiliations:** 1 Mateur Higher School of Agriculture, University of Carthage, Mateur 7030, Tunisia; dhifaliiheb6@gmail.com (I.D.); elgtari.mohamed@gmail.com (M.E.G.); jemmali.bayrem@gmail.com (B.J.); 2 Veterinary Research Institute of Tunisia, Tunis 1006, Tunisia; ouledahmedh@yahoo.fr; 3 National Foundation for the Improvement of the Horse Breed, Sidi Thabet 2020, Tunisia; faten.lasfar@topnet.tn

**Keywords:** Tunisian Arabian horses, microsatellites, genetic diversity, heterozygosity, gene flow, PCA

## Abstract

The genetic diversity and population structure of Tunisian Arabian horses were assessed using highly polymorphic microsatellite markers, which are critical for conservation and breeding programs. Despite the cultural and economic importance of Arabian horses in Tunisia, molecular data supporting their management remain limited. In this study, DNA from 130 horses was genotyped with 17 ISAG-FAO-recommended microsatellites to evaluate diversity within Eastern and Western Arabian lineages and their relationship to Thoroughbreds. Eastern Arabians showed an average of 5.176 alleles per locus, observed heterozygosity of 0.657, expected heterozygosity of 0.677, and a fixation index of 0.028, while Western Arabians displayed 5.941 alleles, heterozygosity values of 0.689 (Ho) and 0.688 (He), and a fixation index of −0.006. Genetic differentiation was low between Eastern and Western Arabians (0.011) but moderate between Eastern Arabians and Thoroughbreds (0.071), with high gene flow within Arabian subpopulations (0.950). Principal component analysis confirmed distinct subpopulations. These findings highlight high genetic diversity in Western Arabians and variable heterozygosity in Eastern Arabians, providing a molecular basis for targeted breeding strategies to preserve genetic traits, control inbreeding, and ensure the long-term sustainability of Tunisian Arabian horse populations.

## 1. Introduction

Genetic diversity is fundamental to the survival and adaptation of animal species, enabling populations to withstand environmental changes and avoid extinction. Variability in genes supports key traits such as disease resistance, reproductive success, and behavioral adaptability, making it a cornerstone of natural selection and conservation biology.

Recent studies have also demonstrated the usefulness of microsatellite markers and related molecular tools for assessing genetic diversity and identifying population structure in equine and livestock species, further highlighting their relevance for conservation genetics [1,2]

Microsatellites have become essential tools for studying genetic diversity and population structure since their adoption in the late 1980s. Their high polymorphism, reproducibility, and relatively low cost make them particularly effective for equine studies [3,4,5]. These markers are widely used to assess genetic variation among breeds, supported by PCR amplification of flanking regions and electrophoretic separation to generate detailed genetic profiles [6].

Despite the advances in molecular tools, little is known about the genetic composition of Tunisian horse breeds, particularly Arabian horses that hold cultural and economic importance. Previous studies using PCR and sequencing approaches have provided preliminary insights [7,8,9], but comprehensive analyses remain limited. This gap highlights the need for molecular investigations to inform sustainable breeding and conservation strategies. Similar molecular investigations conducted in other horse and domestic animal populations have shown how such approaches substantially improve the understanding of genetic structure and support the development of more effective breeding and conservation strategies [10].

The present study applies ISAG-FAO-recommended microsatellites to evaluate the genetic diversity and structure of Tunisian Arabian horses. Specifically, we investigate relationships between Eastern and Western lineages and assess their differentiation from Thoroughbreds. We hypothesize that Western Arabians maintain higher genetic diversity than Eastern Arabians, reflecting differences in breeding history. By addressing these questions, the study aims to provide a molecular foundation for conservation programs and sustainable management of Tunisian Arabian horse populations

## 2. Materials and Methods

### 2.1. Samples and DNA Extraction

A total of 130 horses were included in this study. Among them, 99 were Tunisian Arabian horses registered in the national studbook and classified into two lineages based on pedigree information: 36 Eastern (Oriental-type) Arabians and 63 Western (Occidental-type) Arabians. In addition, 31 English Thoroughbred horses were included as an outgroup to assess inter-population genetic differentiation.

Although sampling was performed to avoid obvious close relatives based on studbook information, no formal pedigree-based or molecular relatedness analysis was conducted. Therefore, some degree of relatedness within each lineage cannot be fully excluded.

Blood samples are collected from the jugular vein of animals and placed into vacuum tubes containing 5 mL vacuum tubes with 20 µL of K3 EDTA (BD Vacutainer®, Becton, Dickinson and Company, Franklin Lakes, NJ, USA).

Genomic DNA was extracted using the PureLink Genomic DNA Extraction Kit (Invitrogen™, Thermo Fisher Scientific, Waltham, MA, USA), which enables rapid and efficient purification of genomic DNA. This kit is specifically designed to isolate high-quality DNA by separating it from other cellular and tissue components.

### 2.2. Microsatellites Used

Microsatellites selected for genotyping the Tunisian Arabian breed to assess intrapopulation genetic variability consist of a panel of 17 markers (Table 1) recommended by the ISAG-FAO Advisory Group for global equine genetic diversity analysis, identification, and parentage verification [11].

### 2.3. DNA Amplification by PCR

Genomic DNA was amplified using multiplex PCR with the Applied Biosystems Equine Genotyping Kit(Applied Biosystems, Foster City, CA, USA), following FAO guidelines. Each PCR reaction contained 50 ng of genomic DNA. PCR conditions included an initial denaturation at 95 °C for 10 min, followed by 30 cycles of denaturation at 95 °C for 30 s, annealing at 60 °C for 30 s, and extension at 72 °C for 1 min, with a final extension step at 72 °C for 1 h to amplify the targeted microsatellites. Each 25 µL reaction mixture contained specific components: 2.5 µL MgCl2 (25 mM), 0.2 µL dNTP (25 mM), 0.5 µL of each primer (25 pmol/µL), 0.2 µL Taq polymerase (5 U/µL), 50 ng DNA, and 2.5 µL of 5X buffer. Amplified products were analyzed by capillary electrophoresis using an ABI Prism 3130 DNA Genetic Analyzer (Applied Biosystems, Foster City, CA, USA), and DNA fragment sizes were determined using GeneScan-500 LIZ with GeneMapper Software (Applied Biosystems, Ver. 4.).

### 2.4. Parameters of Genetic Variability

The level of variability was used to examine the genetic variety within the studied population. The Hardy–Weinberg’s law was applied to determine whether a population was in equilibrium. We calculated parameters to characterize genetic diversity. Direct comparison of allele frequencies is challenging when considering the genetic heterogeneity within populations. Utilizing the GenAlex program (version 6.2), the following were calculated.

#### 2.4.1. The Intra-Population Parameters

##### Allelic Frequency

Allelic frequency is the fundamental parameter used to describe and analyze genetic variability within a population [21]. Thus, the frequency of an allele in a sample is equal to two times the range of homozygous genotypes for that allele (every homozygote consists of copies of the allele), plus the range of heterozygous genotypes containing that allele (each heterozygote incorporates one copy), divided by way of twice the overall range of people within the pattern (because every individual carry two alleles at this locus) [22].

The formula to calculate the frequency (Pi) of allele i at locus k in population x is as follows:

$$Pikx=\frac{2*\left(nii\right)+ni}{2*N}$$

nii represents the count of individuals homozygous for allele i at locus k.

ni denotes the count of individuals heterozygous for allele i at locus.

N is the total number of individuals typed at locus k.

With:

$$\sum_{i=1}^{lk}Pik=1$$

ik represents the number of alleles at locus k.

##### The Rate of Heterozygosity

The most commonly used indicator of intra-population genetic variability, as generalized by [23] and also referred to as the diversity index, is the probability that two random genes represent different alleles. It is equivalent to the expected heterozygosity rate (H) under the assumptions of Hardy–Weinberg equilibrium [24].

The heterozygosity rate corresponds to the proportion of heterozygous individuals at a given locus, and the overall heterozygosity of a population is obtained by averaging these values across all loci studied.

Ho=1/N∑Hi

With:

N: the total number of loci studied.

Hi: the heterozygosity at locus i.

A good estimation of genetic variability is obtained through the population heterozygosity rate, as long as individuals within the population reproduce randomly.

##### Polymorphism Rate of Microsatellite Markers

Another way to assess the genetic variability of a population is by considering the number of alleles existing for the analyzed markers. A large number of alleles indicate greater diversity. A marker is considered highly polymorphic when it has at least two alleles, and the frequency of the most common allele is equal to or less than 0.95 [25].

##### Fixation Index

The fixation index (Fis), also known as the inbreeding coefficient, is calculated from the difference between the proportion of individuals found in the heterozygous state (ho) and the expected heterozygosity rate (he) under the assumption of equilibrium [26], according to the following formula:

$$Fis=\frac{he-ho}{he}=1-\frac{ho}{he}$$

With:

ho: observed heterozygosity.

he: expected heterozygosity, calculated from allele frequencies under the Hardy–Weinberg equilibrium assumption.

This parameter reflects the differentiation of individuals within populations:

Fis = 1 signifies complete fixation (as in the case of self-fertilization).

0 < F < 1 indicates heterozygote deficit.

F = 0 indicates a population in Hardy–Weinberg equilibrium.

Fis < 0 indicates excess heterozygosity.

##### Observed and Effective Number of Alleles (Na and Ne)

The observed number of alleles (Na) corresponds to the total number of alleles detected at each locus within a population. The effective number of alleles (Ne) represents the number of equally frequent alleles required to generate the observed level of genetic diversity.

Ne is calculated as:

$$Ne=\frac{1}{\sum f_{i}^{2}}$$

where $f_{i}$ is the frequency of allele i.

#### 2.4.2. Inter-Population Parameters

##### Wright’s F-Statistics

The most classical method of population characterization, and perhaps the oldest, is that of fixation indices proposed by [27].

The F-statistics enable the description of population structure, the distribution of genetic variability between and within populations by estimating the standardized variance of allelic frequencies among subpopulations [28].

FST, also known as fixation index, indicates the reduction in heterozygosity within subpopulations due to differences in average allelic frequencies. It provides information about population differentiation and subdivision effects. It represents the correlation between alleles within a subpopulation relative to all subpopulations. It takes a value of zero when all subpopulations have the same allelic frequencies and are in Hardy–Weinberg equilibrium. In cases of differentiation, its value ranges from 0 to 1 [29].

0 < FST < 0.05: Weak differentiation

0.05 < FST < 0.15: Moderate differentiation

0.15 < FST < 0.25: Significant differentiation

F > 0.25: Very significant differentiation

FIT represents the reduction in heterozygosity between an individual and the theoretical overall population, considering both within- and between-subpopulation effects. It integrates the contributions of Fis (inbreeding within subpopulations) and FST (differentiation among subpopulations) according to the relationship:

(1−FIT)=(1−FIS)(1−FST)

A positive FIT indicates an overall deficit of heterozygotes in the global population, whereas negative values reflect an excess of heterozygosity. FIT therefore provides a global assessment of deviation from Hardy–Weinberg equilibrium across all subpopulations combined.

##### Nei’s Genetic Distance

In 1972, Nei proposed the most commonly used genetic distance, which is defined as follows:

D = −Log I

With I = I′/r, where r is the number of loci, and I′ is the similarity index defined at each locus.

$$I^{\prime }=\frac{J12}{J11J22}$$

Indices 1 and 2 represent populations 1 and 2, respectively.

##### Dendrogram

The Neighbor-Joining [24] tree-building method has been used to construct dendrograms from the distance matrix calculated according to Reynolds [25].

##### Gene Flow (Nm)

Gene flow is defined as the exchange of multiple genes or their alleles between different related populations.

Gene flow represents the number of migrants exchanged between populations per generation. It is estimated from Wright’s fixation index using the formula: Nm = (1 − FST)/(4 FST). A high Nm value indicates substantial gene exchange and low genetic differentiation, whereas a low Nm value suggests restricted migration and stronger population subdivision. In this study, Nm was calculated for each pair of populations using their corresponding FST values to assess the level of connectivity among groups.

## 3. Results

### 3.1. Intra-Population Genetic Variability

#### 3.1.1. Polymorphism Rate

A locus is considered polymorphic at a 5% threshold if it exhibits at least two different alleles. In our study, the polymorphism rate reached 100%, indicating that all analyzed loci are polymorphic.

#### 3.1.2. Total Number of Alleles and Allelic Richness

Allelic richness, defined as the number of alleles at a given locus, is influenced by sample size, since the probability of detecting new alleles increases with the number of individuals analyzed [30].

The comparative values reported for Syrian, Iranian, Saudi, Polish, Shagya, USA-Egyptian, USA-Saudi, and Davenport Arabian horses are not from our dataset but were extracted from previously published microsatellite studies mainly [26]. These published datasets were used solely to contextualize the genetic diversity of Tunisian horses.

The allelic counts reported for Eastern and Western Arabians correspond to the 99 purebred Arabians included in the study. The remaining 31 individuals represent the Thoroughbred reference population used for genetic distance, F-statistics, and PCA analyses.

For Eastern Arabian horses, a total of 88 distinct alleles were identified across 17 microsatellites in 36 individuals. The average number of alleles per locus was 5.176 ± 0.246, confirming that each locus possessed more than two alleles. The microsatellites ASB2 and LEX3 displayed the highest polymorphism (≈7 alleles), whereas VHL20, HTG7, HTG4, AHT4, and HTG6 were the least polymorphic (4 alleles).

Among Syrian non-registered horses, the highest genetic diversity was recorded, with an average of 8.47 ± 0.59 alleles, while registered Syrian horses had slightly lower values (6.47 ± 0.38 alleles). Iranian Arabian horses showed intermediate diversity (5.93 ± 0.37 alleles), and Saudi registered horses presented relatively low diversity (5.13 ± 0.31 alleles). Tunisian Eastern Arabian horses showed reduced values (5.176 ± 0.246 alleles).

For Western Arabian horses, 101 alleles were detected in 63 individuals, with an average of 5.941 ± 0.388 alleles per locus. The microsatellite ASB2 was the most polymorphic (≈9 alleles), whereas HTG6 was the least polymorphic (3 alleles). Within Western populations, Polish Arabian and Shagya Arabian horses displayed high levels of genetic diversity (5.67 ± 0.40 and 4.93 ± 0.30 alleles, respectively), while USA-Saudi and USA-Egyptian horses exhibited lower values (4.40 ± 0.31 and 4.00 ± 0.26, respectively). Davenport horses showed the lowest diversity (3.00 ± 0.31 alleles). Interestingly, Tunisian Western Arabian horses demonstrated relatively high genetic diversity (5.941 ± 0.388 alleles), comparable to Polish and Shagya Arabians.

#### 3.1.3. Allelic Frequency

Analyzing the allelic frequency spectrum provides insight into the genetic variability within the studied population.

For Eastern Arabian Horses, examining the summarized allelic frequencies allows for the identification of abundant alleles and rare alleles, which exhibit low frequencies.

**Figure 1:**
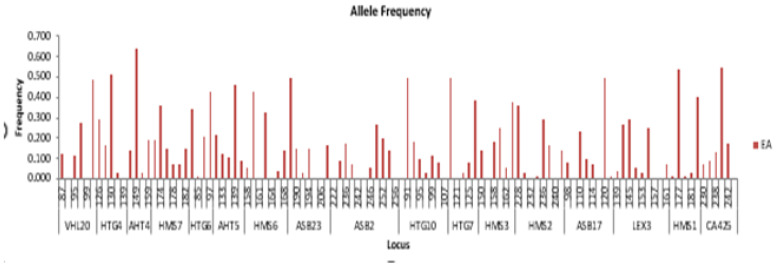
Allelic frequencies of eastern Arabian horses.

According to our study, the distribution of alleles for the analyzed microsatellites varies from 4 alleles for microsatellites VHL20, HTG7, HTG4, AHT4, and HTG6 to 7 alleles for microsatellites ASB2 and LEX3 (Figure 1).

Allelic frequencies calculated for each locus vary between 0.014 for alleles of sizes 234, 128, 175, 179, and 85 base pairs, respectively, for the microsatellites HMS2, ASB17, HMS1, HMS1, and HTG6, to 0.639 for the allele of size 149 base pairs of microsatellites AHT4.

A total of 3 private alleles were identified in this study, all with frequencies below 1.4% (Table 2).

**Figure 2:**
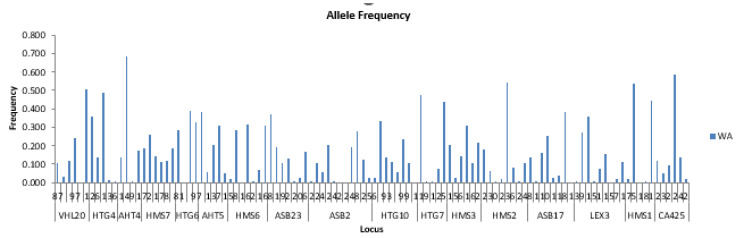
Allelic frequencies of western Arabian horses.

For Western Arabian horses, allelic frequencies ranged from 0.008 (HTG4-139, AHT4-151, HMS6-164, ASB23-202, ASB2-222 and 242, HTG7-121 and 123, ASB17-108, LEX3-103 and 151, HMS1-181 bp) to 0.683 (AHT4-149 bp) (Figure 2).

Table 3 shows the most abundant allelic frequencies as well as the lowest ones for each amplified region. These frequencies vary depending on the inheritance of the total population.

Private alleles in this population were also generally rare (<0.8%), although specific alleles exhibited higher frequencies: HMS1-178 (northern population), and ASB23-206, ASB2-256, HMS3-156, HMS2-240, ASB17-118, CA425-244 (Western Purebred Arabian population) (Table 4).

Overall, Western Arabian horses displayed slightly higher general allelic frequencies compared to Eastern Arabian horses. The majority of private alleles in both populations occurred at low frequencies, indicating limited distribution across individuals.

#### 3.1.4. Heterozygosity Rate

Observed (Ho) and expected (He) heterozygosity rates were calculated for each locus under the assumption of Hardy–Weinberg equilibrium to assess genetic polymorphism.

For Eastern Arabian horses, the highest observed heterozygosity was 0.857 at locus HMS6, while the lowest was 0.472 at loci AHT4 and LEX3. The mean observed heterozygosity was 0.657 ± 0.029 (Table 5).

In Western Arabian horses, the highest Ho was 0.873 at HMS7, and the lowest was 0.339 at LEX3. The mean observed heterozygosity was 0.689 ± 0.035, and the mean expected heterozygosity was 0.688 ± 0.025, with values ranging from 0.485 (AHT4) to 0.818 (HMS7) (Table 6).

In both populations, approximately 65–69% of horses were heterozygous across the 17 microsatellites studied, indicating high intra-population heterogeneity. Comparisons with previous studies (Table 7) show that Syrian non-registered horses had the highest observed Ho (0.72) relative to He (0.75), while Tunisian horses exhibited slightly lower Ho (0.657) than He (0.677). Polish and Shagya Arabians displayed observed heterozygosity close to or slightly higher than expected (0.69 and 0.68 vs. 0.68 and 0.69), whereas USA-Egyptian and Davenport horses had lower observed than expected heterozygosity, suggesting possible genetic bottlenecks.

#### 3.1.5. Allelic Diversity: Observed (Na) and Effective (Ne) Alleles

The observed number of alleles (Na) represents the total number of alleles present in a population for a given locus, whereas the expected number of alleles (Ne) reflects the number of alleles that contribute most to genetic diversity, also known as the effective number of alleles.

For Eastern Arabian horses, Na for the 17 analyzed microsatellites ranged from 4 alleles (VHL20, HTG4, AHT4, HTG6, HTG7) to 7 alleles (ASB2, LEX3), with an average of 5.176 ± 0.246. The expected number of alleles (Ne) ranged from 2.146 (AHT4) to 5.581 (ASB2), with an average of 3.295 ± 0.216 (Table 8).

For Western Arabian horses, Na ranged from 3 alleles (HTG6) to 9 alleles (ASB2), with an average of 5.941 ± 0.388, while Ne ranged from 1.943 (AHT4) to 5.490 (HMS7), averaging 3.534 ± 0.271. Across all loci in both populations, the observed number of alleles exceeded the expected number, indicating substantial genetic heterogeneity.

#### 3.1.6. Fixation Index (Fis)

The fixation index (Fis) measures the deviation between observed heterozygosity (Ho) and expected heterozygosity (He) in populations that deviate from Hardy–Weinberg equilibrium (HWE). If Fis = 0, the population is in HWE; negative values indicate an excess of heterozygosity, while positive values indicate a heterozygote deficit.

For the Eastern Purebred Arabian population, Fis values ranged from −0.255 at locus HMS6 (excess of heterozygotes) to 0.389 at locus LEX3 (heterozygote deficit), with an average of 0.028 (Table 9).

Ten loci (HTG4, AHT4, HMS7, HTG6, ASB2, HTG10, HMS2, ASB17, LEX3, HMS1) displayed positive Fis values (0 < Fis < 1), suggesting heterozygote deficiency, while the remaining loci (VHL20, AHT5, HMS6, ASB23, HTG7, HMS3, CA425) exhibited negative values, consistent with heterozygote excess.

Departures from Hardy–Weinberg equilibrium observed at several loci may partly reflect residual relatedness among sampled individuals, in addition to potential effects of breeding structure and allele frequency distribution.

For the Western Arabian population, Fis values ranged from −0.250 at locus HMS1 to 0.553 at locus LEX3, with an average of −0.006 (Table 9). Five loci (HTG4, AHT5, HTG7, HMS2, LEX3) showed positive Fis values, confirming heterozygote deficiency, whereas the remaining loci displayed negative values, indicating heterozygote excess. Similarly, all loci deviated from zero, indicating that this population is also not in Hardy–Weinberg equilibrium.

### 3.2. Inter-Population Genetic Variability

#### 3.2.1. Parameters of Population Differentiation

The classical method for characterizing population structure is based on Wright’s fixation indices [26,27]. These indices allow the distribution of genetic variability to be described both within and among populations by estimating the standardized variance of allelic frequencies. Wright’s F-statistics include: (i) the overall fixation index (FIT), (ii) the inbreeding coefficient within subpopulations (FIS), and (iii) the genetic differentiation among subpopulations (FST).

Table 10 presents the FST values among the three studied populations (Eastern Arabian, Western Arabian, and Thoroughbred horses).

The lowest FST value (0.011) was observed between Eastern and Western Arabian horses, indicating minimal differentiation. The highest value (0.071) was observed between Eastern Arabians and Thoroughbreds, exceeding Wright’s 0.05 threshold for moderate genetic differentiation.

For the three populations combined, the mean FST was 0.058 (Table 11), suggesting that 5.8% of the total genetic variation is due to differences between populations, while 94.2% occurs within populations.

FIS values ranged from −0.100 (HMS6) to 0.396 (LEX3), with a mean of 0.018 (±0.027), whereas FIT values ranged from −0.027 (HMS6) to 0.414 (LEX3), with a mean of 0.076 (±0.025). Overall, the mean FIT indicates a 7.6% deficit of heterozygotes across all loci.

#### 3.2.2. Genetic Differentiation Based on Nei’s Distance

Genetic distance values among the three studied populations are presented in Table 12.

The lowest genetic distance (0.052) was observed between Eastern and Western Arabian horses, indicating that 94.8% of the studied microsatellite markers (STRs) are shared between these two populations.

Conversely, the highest genetic distance (0.374) was recorded between Eastern Arabian and Thoroughbred horses, suggesting that only 62.6% of the studied microsatellite markers (STRs) are similar. An intermediate value of 0.243 was obtained between Western Arabians and Thoroughbreds, corresponding to 75.7% genetic similarity.

#### 3.2.3. Analysis of Genetic Exchange (Nm) Between Populations

Gene flow (Nm) values between the studied populations are shown in Table 13.

The highest level of gene flow (0.950) was observed between Eastern and Western Arabian horses, indicating substantial genetic exchange between these two populations. By contrast, the lowest Nm value (0.688) was found between Eastern Arabians and Thoroughbreds, suggesting limited gene flow. An intermediate value of 0.784 was recorded between Western Arabians and Thoroughbreds.

#### 3.2.4. Principal Component Analysis (PCA)

**Figure 3:**
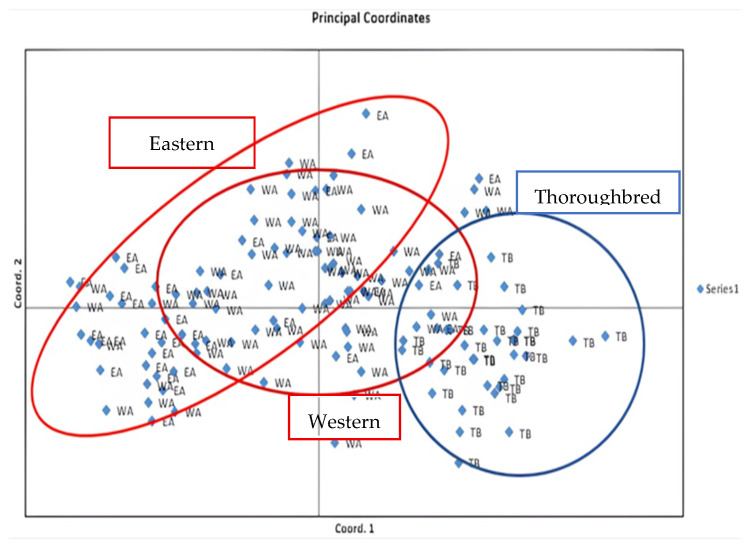
Principal Component Analysis (PCA). EA, Eastern Arabian; WA, Western Arabian; TB, Thoroughbred.

All observed and expected values of the genetic diversity parameters were included in a Principal Component Analysis (PCA) (Figure 3).

The first three principal axes derived from allelic frequencies explained 64.29% of the total inertia, with the first axis accounting for 33.84% of the variance, the second axis for 16.43%, and the third axis for 14.02%. The distribution of the populations in the PCA plot highlights three distinct groups: Purebred Arabian horses of Eastern origin (EA), Purebred Arabian horses of Western origin (WA), and Thoroughbreds (TB).

## 4. Discussion

The consistently high polymorphism rate across loci confirms the effectiveness of microsatellites as reliable markers for evaluating genetic diversity. Variations in allelic richness among populations are consistent with previous findings [30], which emphasized that both the number of loci analyzed and the sample size strongly influence the total number of alleles detected. The elevated allelic richness observed in Syrian and Polish Arabians reflects a broad genetic background and less restrictive breeding practices, whereas the reduced values in Saudi, Egyptian, and Davenport horses suggest narrower genetic pools shaped by selective breeding. The slightly higher diversity recorded in Western compared to Eastern Arabian horses further indicates that historical crossbreeding and the introduction of Western bloodlines particularly in Tunisia have contributed to enhanced genetic variability. These results highlight the importance of maintaining and conserving genetic diversity in horse populations through sustainable breeding and conservation programs. These observations are in accordance with recent findings in other equine and livestock populations, where microsatellite-based analyses have proven effective in characterizing genetic variation and guiding conservation decisions [1,10].

The variation in allelic frequencies reflects both population structure and the influence of breeding practices. Rare alleles (<1.4% in Eastern, <0.8% in Western populations) indicate the presence of low genetic variants specific to each population, thereby contributing to overall genetic diversity [30]. Differences between Eastern and Western Arabian horses suggest that Western populations may have undergone selective pressures or historical breeding programs favoring slightly higher allelic frequencies, while maintaining a lower prevalence of private alleles exceeding 0.8%. These patterns highlight population-specific adaptations and underscore the importance of conserving rare alleles to preserve genetic diversity within horse breeds.

Observed and expected heterozygosity values further reflect differences in allele number across loci, confirming substantial genetic diversity in both Eastern and Western Arabian populations. A slight deficit of heterozygosity in Tunisian horses may indicate historical breeding practices or selective pressures that reduced genetic variation. By contrast, higher observed heterozygosity in Syrian non-registered horses and Polish/Shagya Arabians reflects broader genetic backgrounds and less restrictive breeding management [30]. Western Arabian horses maintain consistently high heterozygosity across loci, whereas Eastern Arabians show greater variation, reflecting localized breeding strategies and potential founder effects. Conserving heterozygosity remains critical to maintaining genetic health and adaptive potential in Arabian horse populations.

Comparable trends were reported in other domestic species, where the relationship between breeding history, heterozygosity levels, and genetic structure was similarly highlighted through microsatellite analyses [2].

The excess of the observed number of alleles (Na) over the effective number of alleles (Ne) in both Eastern and Western Arabian horses indicates a high level of genetic diversity. Western populations display slightly greater overall diversity, with both observed and expected average allele numbers exceeding those of Eastern horses. Locus-to-locus variation highlights the complex genetic structure unique to each population, likely shaped by historical breeding programs, selective mating strategies, and the introduction of specific lineages over time [30]. Preserving this genetic diversity is essential for safeguarding adaptive potential and reducing the risk of inbreeding in future breeding programs.

Because relatedness between individuals was not formally tested, deviations from Hardy–Weinberg equilibrium and some FIS patterns should be interpreted with caution, as undetected in relationships within the lineage groups may contribute to these results.

Fixation index (Fis) results demonstrate further differentiation between populations. In Eastern Arabian horses, the moderately positive mean Fis (0.028) reflects a slight deficit of heterozygotes, consistent with possible inbreeding or selection for homozygotes at certain loci. Conversely, Western Arabians exhibited a slightly negative mean Fis (–0.006), suggesting a subtle excess of heterozygotes and therefore marginally higher genetic diversity. These findings align with previous studies: [30] reported average Fis values of 0.037 in Syrian non-registered Arabian horses, –0.007 in Syrian registered horses, 0.017 in Iranian Arabians, and 0.028 in Tunisian Arabians. The Eastern population analyzed here shows heterozygosity levels comparable to Tunisian horses, while the Western population resembles Syrian registered horses with a negative fixation index, reflecting greater variability. Locus-specific deviations were also observed, with HMS6 (Eastern) and HMS1 (Western) showing the strongest excess of heterozygotes, whereas LEX3 consistently indicated a heterozygote deficit in both groups. It is important to note that LEX3 is an X-linked marker, and males are hemizygous rather than homozygous. Because sex was not incorporated into the computation of heterozygosity and fixation indices, the observed heterozygote deficit and the high FIT value at this locus may be partly influenced by its sex-linked inheritance pattern. Thus, LEX3-related deviations should be interpreted with caution. These patterns may reflect historical breeding practices, genetic drift, or the presence of null alleles, all of which shape Arabian horse diversity.

Genetic differentiation indices further support these findings. The low FST value (0.011) between Eastern and Western Arabians suggests substantial gene flow and shared ancestry, consistent with their close historical and geographical relationship. In contrast, the higher FST (0.071) observed between Eastern Arabians and Thoroughbreds reflects moderate differentiation, likely driven by distinct breeding histories and selection pressures. The overall average FST of 0.058 indicates moderate genetic differentiation among the three populations, consistent with Wright’s classification [16]. Similarly, the heterozygosity deficit reflected by the positive average FIT (0.076) suggests some degree of non-random mating or selection for homozygous genotypes, while keeping in mind that the extreme FIT at LEX3 (0.414) is potentially driven by its X-linked nature. Conversely, loci such as HMS6, with negative FIS and FIT values, highlight an excess of heterozygotes, likely resulting from balancing selection or the presence of null alleles.

Genetic distance values further corroborate these patterns. Eastern and Western Arabian horses displayed the smallest distance (0.052), confirming their close relationship and possible historical gene flow, with 94.8% of genes being shared. In contrast, the greatest distance (0.374) was observed between Eastern Arabians and Thoroughbreds, indicating substantial divergence (62.6% similarity), consistent with the closed breeding practices of the latter. An intermediate distance (0.243) between Western Arabians and Thoroughbreds suggests some degree of shared ancestry or historical crossbreeding. Overall, the observed genetic distances are consistent with the FST results, confirming that Arabian subpopulations are more closely related to each other than to Thoroughbreds.

Gene flow (Nm) estimates also support this conclusion. The highest value (0.950) was found between Eastern and Western Arabian horses, confirming their strong genetic connectivity. By contrast, lower values were observed between Eastern Arabians and Thoroughbreds (0.688), reflecting the genetic isolation of the latter, while the intermediate value between Western Arabians and Thoroughbreds (0.784) may be explained by occasional historical introgression or shared ancestral contributions. Collectively, these results indicate that the Western Arabian population has exerted a more pronounced influence on the genetic structure of the Eastern population, whereas Thoroughbreds remain comparatively distinct.

Finally, the Principal Component Analysis (PCA) confirmed the existence of two distinct subpopulations (Eastern and Western) within the Arabian breed, reflecting the impact of specific breeding practices and initial differences in origin on genetic structure. The PCA also revealed strong genetic similarity between the two Arabian subgroups, consistent with historical interbreeding and shared ancestry. In contrast, Thoroughbreds were clearly separated from Eastern Arabians, with only a minority of individuals clustering closer to Western Arabians. This emphasizes both the divergence between Thoroughbreds and Arabians and the occasional genetic contribution of Western Arabians to Thoroughbred populations.

## 5. Conclusions

During the molecular analysis and evaluation of genetic variability within the Tunisian Arabian horse population, 17 microsatellites were carefully chosen for their relevance. Results show that the parameter of the number of alleles per locus (Na) in both the Eastern and Western populations have a high rate, with observed values consistently exceeding the expected values, highlighting the significant genetic diversity of the purebred Arabian population in Tunisia. The average fixation index is negative (−0.06) for the Western population, confirming the importance of genetic diversity within the studied population and indicating low consanguinity. Conversely, for the Eastern population, the average fixation index is (0.028), confirming a deficit of heterozygosity compared to what would be expected under Hardy–Weinberg equilibrium. Genetic distance results indicate that 94.8% of the studied microsatellite loci show similarity between the Eastern Arabian and Western Arabian populations, while 62.6% of the loci analyzed demonstrate similarity between the Eastern Arabian and English Thoroughbred populations, and 75.7% of the studied loci exhibit similarity between the Western Arabian and English Thoroughbred populations. Additionally, PCA confirms that the Western Arabian population clusters somewhat with the English Thoroughbred population, albeit to a lesser extent compared to its similarity with the Eastern Arabian population.

## Figures and Tables

**Table 1:** Characteristics of the 17 microsatellites uses from the Stock Marks (Applied Biosystems, Foster City, CA, USA).

Loci	Microsatellite Sequences	Length Size (bp)	References
AHT4	5′AACCGCCTGAGCAAGGAAGT	144–164	[12]
	3′CCCAGAGAGTTTACCCT		
AHT5	5′ACGGACACATCCCTGCCTGC	126–144	[13]
	3’GCAGGCTAAGGAGGCTCAGC		
ASB2	5′CCACTAAGTGTCGTTTCAGAAGG	216–250	[14]
	3′CACAACTGAGTTCTCTGATAGG		
ASB17	5′ACCATTCAGGATCTCCACCG	87–129	[14]
	3′GAGGGCGGTACCTTTGTACC		
ASB23	5′GAGGGCAGCAGGTTGGGAAGG	175–211	[15]
	3′ACATCCTGGTCAAATCACAGTCC		
CA425	5′AGCTGCCTCGTTAATTCA	226–246	[16]
	3′CTCATGTCCGCTTGTCTC		
HMS1	5′CATCACTCTTCATGTCTGCTTGG	170–186	[17]
	3′TTGACATAAATGCTTATCCTATGGC		
HMS2	5′CTTGCAGTCGAATGTGTATTAAATG	222–248	[17]
	3′ACGGTGGCAACTGCCAAGGAAG		
HMS3	5′CCATCCTCACTTTTTCACTTTGTT	148–170	[17]
	3′CCAACTCTTTGTCACATAACAAGA		
HMS6	5′GAAGCTGCCAGTATTCAACCATTGG	151–169	[17]
	3′CTCCATCTTGTGAAGTGTAACTCA		
HMS7	5′TGTTGTTGAAACATACCTTGACTGT	165–185	[17]
	3′CAGGAAACTCATGTTGATACCATC		
HTG4	5′CTATCTCAGTCTTGATTGCAGGAC	127–139	[12]
	3′CTCCCTCCCTCCCTCTGTTCTC		
HTG6	5′GTTCACTGAATGTCAAATTCTGCT	84–102	[12]
	3′CCTGCTTGGAGGCTGTGATAAGAT		
HTG7	5′CCTGAAGCAGAACATCCCTCCTTG	118–128	[18]
	3’ATAAAGTGTCTGGGCAGAGCTGCT		
HTG10	5’TTTTTATTCTGATCTGTCCACATTT	95–115	[18]
	3’CAATTCCCGCCCCACCCCCGGCA		
VHL20	5′CAAGTCCTCTTACTTGAAGACTAG	87–105	[19]
	3′AACTCAGGGAGAATCTTCCTCAG		
LEX3	5′ACATCTAACCAGTGCTGAGACT 3′GAAGGAAAAAAAGGAGGAAGAC	142–164	[20]

**Table 2:** Private alleles detected in Eastern Arabian horses.

Microsatellite	Base Pairs	Allelic Frequencies
HMS1	179	0.014
ASB17	128	0.014
HTG6	85	0.014

**Table 3:** Lowest-frequency alleles in Western Arabian horses.

Microsatellite	Base Pairs	Allelic Frequencies
ASB2	222	0.008
	242	0.008
HTG4	139	0.008
AHT4	151	0.008
HMS6	164	0.008
ASB23	202	0.008
HTG7	121	0.008
	123	0.008
ASB17	108	0.008
LEX3	103	0.008
	151	0.008
HMS1	181	0.008

**Table 4:** Private alleles detected in Western Arabian horses.

Microsatellite	Base Pairs	Allelic Frequencies
HTG4	139	0.008
HMS6	164	0.008
ASB23	202	0.008
ASB23	206	0.024
ASB2	242	0.008
ASB2	256	0.025
HTG7	121	0.008
HMS3	156	0.024
HMS2	240	0.009
ASB17	108	0.008
ASB17	118	0.040
CA425	244	0.017

**Table 5:** The observed heterozygosity rate (Ho) and the expected heterozygosity rate (He) in Eastern Arabian horses.

Microsatellite	Ho	He
VHL20	0.694	0.659
HTG4	0.611	0.622
AHT4	0.472	0.534
HMS7	0.750	0.775
HTG6	0.639	0.650
AHT5	0.750	0.703
HMS6	0.857	0.683
ASB23	0.750	0.675
ASB2	0.800	0.821
HTG10	0.528	0.688
HTG7	0.611	0.591
HMS3	0.806	0.742
HMS2	0.667	0.736
ASB17	0.611	0.673
LEX3	0.472	0.772
HMS1	0.500	0.543
CA425	0.657	0.647
Average	0.657	0.677

**Table 6:** The observed heterozygosity rate (Ho) and the expected heterozygosity rate (He) In Western Arabian horses.

Microsatellite	Ho	He
VHL20	0.698	0.659
HTG4	0.619	0.620
AHT4	0.492	0.485
HMS7	0.873	0.818
HTG6	0.746	0.661
AHT5	0.654	0.711
HMS6	0.783	0.720
ASB23	0.823	0.768
ASB2	0.852	0.814
HTG10	0.855	0.790
HTG7	0.540	0.577
HMS3	0.790	0.785
HMS2	0.586	0.651
ASB17	0.758	0.747
LEX3	0.339	0.758
HMS1	0.650	0.520
CA425	0.661	0.614
Average	0.689	0.688

**Table 7:** Comparison of observed and expected mean heterozygosity.

Oriental Arabian	Source	The Average Observed Heterozygosity	The Average Expected Heterozygosity
Saudi Arabian	[30]	0.68 (±0.03)	0.68 (±0.03)
Syrian registered	[30]	0.70 (±0.03)	0.69 (±0.03)
Syrian nonregistered	[30]	0.72 (±0.02)	0.75 (±0.02)
Iranian Arabian	[30]	0.70 (±0.02)	0.71 (±0.02)
Tunisia Eastern	The current study	0.657 (±0.029)	0.677 (±0.019)
Tunisia western	The current study	0.689 ± 0.035	0.688 (±0.025)

**Table 8:** Observed and expected number of alleles (Na and Ne).

Microsatellite	Na (Eastern Arabian Horses)	Ne (Eastern Arabian Horses)	Na (Western Arabian Horses)	Ne (Western Arabian Horses)
VHL20	4	2.929	5	2.937
HTG4	4	2.648	5	2.628
AHT4	4	2.146	4	1.943
HMS7	6	4.454	6	5.490
HTG6	4	2.861	3	2.952
AHT5	5	3.372	5	3.460
HMS6	5	3.153	6	3.566
ASB23	5	3.075	7	4.309
ASB2	7	5.581	9	5.385
HTG10	6	3.204	7	4.754
HTG7	4	2.445	5	2.367
HMS3	5	3.874	6	4.659
HMS2	6	3.795	8	2.869
ASB17	6	3.057	7	3.949
LEX3	7	4.393	8	4.134
HMS1	5	2.189	4	2.084
CA425	5	2.832	6	2.591
Total	88	56.008	101	60.078
Average	5.176	3.295	5.941	3.534

**Table 9:** Fixation index (FIS) values for Eastern and Western Arabian horses.

Microsatellite	Fixation Index for Eastern Arabian Horses	Fixation Index for Western Arabian Horses
VHL20	−0.054	−0.059
HTG4	0.018	0.001
AHT4	0.116	−0.014
HMS7	0.033	−0.067
HTG6	0.018	−0.128
AHT5	−0.066	0.080
HMS6	−0.255	−0.089
ASB23	−0.111	−0.071
ASB2	0.025	−0.047
HTG10	0.233	−0.083
HTG7	−0.034	0.065
HMS3	−0.086	−0.006
HMS2	0.095	0.100
ASB17	0.092	−0.015
LEX3	0.389	0.553
HMS1	0.080	−0.250
CA425	−0.016	−0.076
Average	0.028	−0.006

**Table 10:** FST values between pairs of the three populations.

Population	Western	Eastern	Thoroughbreds
Western	0.000		
Eastern	0.011	0.000	
Thoroughbreds	0.049	0.071	0.000

**Table 11:** F-statistic parameters for the three populations.

Microsatellite	FIS	FIT	FST
VHL20	−0.022	0.073	0.093
HTG4	0.025	0.073	0.050
AHT4	0.011	0.100	0.090
HMS7	−0.051	0.001	0.050
HTG6	−0.011	0.064	0.074
AHT5	0.002	0.049	0.048
HMS6	−0.100	−0.027	0.066
ASB23	−0.041	0.007	0.046
ASB2	0.017	0.045	0.029
HTG10	0.045	0.095	0.053
HTG7	−0.044	0.084	0.123
HMS3	−0.061	−0.005	0.053
HMS2	0.140	0.212	0.084
ASB17	0.029	0.100	0.073
LEX3	0.396	0.414	0.030
HMS1	−0.001	0.020	0.020
CA425	−0.022	−0.008	0.015
Average	0.018 (±0.027)	0.076 (±0.025)	0.058 (±0.007)

**Table 12:** Genetic distance.

Population	Western	Eastern	Thoroughbreds
Western	0.000		
Eastern	0.052	0.000	
Thoroughbreds	0.243	0.374	0.000

**Table 13:** Gene flow.

Population	Western	Eastern	Thoroughbreds
Western	-		
Eastern	0.950	-	
Thoroughbreds	0.784	0.688	-

## Data Availability

Data are only available upon request due to restrictions, e.g., privacy or ethical reasons, and are available from the corresponding author with the permission of the National Foundation for the Improvement of Horse Breeds.
